# ReSTiNet: An Efficient Deep Learning Approach to Improve Human Detection Accuracy

**DOI:** 10.1016/j.mex.2022.101936

**Published:** 2022-12-02

**Authors:** Shahriar Shakir Sumit, Dayang Rohaya Awang Rambli, Seyedali Mirjalili, M. Saef Ullah Miah, Muhammad Mudassir Ejaz

**Affiliations:** a Department of Computer & Information Sciences, Universiti Teknologi PETRONAS (UTP), Seri Iskandar, Perak 32610, Malaysia; b Centre for Artificial Intelligence Research and Optimization, Torrens University Australia, Fortitude Valley, QLD 4006, Australia; c Yonsei Frontier Lab, Yonsei University, Seoul, South Korea; d Faculty of Computing, College of Computing and Applied Sciences, Universiti Malaysia Pahang, Pekan 26600, Malaysia; e Electrical & Electronics Engineering, Universiti Teknologi PETRONAS (UTP), Seri Iskandar, Perak 32610, Malaysia; f University Research and Innovation Center, Obuda University, 1034 Budapest, Hungary

**Keywords:** Computer vision, Object detection, Human detection, Tiny convolutional neural network, Low memory devices, ReSTiNet

## Abstract

Human detection is an important task in computer vision. It is one of the most important tasks in global security and safety monitoring. In recent days, Deep Learning has improved human detection technology. Despite modern techniques, there are very few optimal techniques to construct networks with a small size, deep architecture, and fast training time while maintaining accuracy. ReSTiNet is a novel small convolutional neural network that overcomes the problems of network size, detection speed, and accuracy. The developed ReSTiNet contains fire modules by evaluating their number and position in the network to minimize the model parameters and network size. To improve the detection speed and accuracy of ReSTiNet, the residual block within the fire modules is carefully designed to increase the feature propagation and maximize the information flow in the network. The developed approach compresses the well-known Tiny-YOLO architecture while improving the following features: *(i)* small model size, *(ii)* faster detection speed, *(iii)* resolution of overfitting, and *(iv)* better performance than other compact networks such as SqueezeNet and MobileNet in terms of mAP on the Pascal VOC and MS COCO datasets.

ReSTiNet is 10.7 MB, five times smaller than Tiny-YOLO. On Tesla k80, mAP is 27.3% for MS COCO and 63.74% for PASCAL VOC. The validation of the proposed ReSTiNet model has been done on INRIA person dataset using the Tesla K80.

• All the necessary steps, algorithms, and mathematical formulas for building the net- work are provided.
• The network is small in size but has a faster detection speed with high accuracy.

## Graphical abstract

**Figure:**
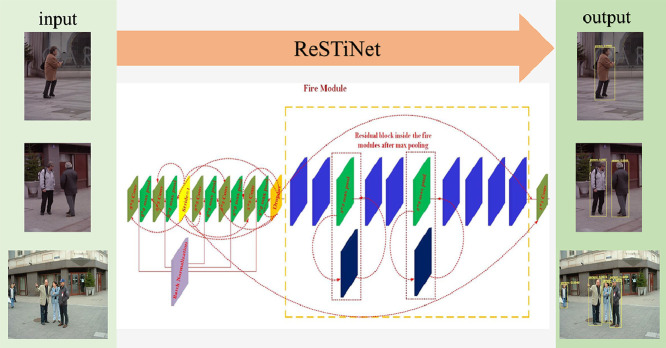

Specifications table

**Table:** 

Subject area:	Computer Science
More specific subject area:	Computer Vision: Object Detection
Name of your method:	ReSTiNet
Name and reference of original method:	Tiny-YOLO [1], SqueezeNet [2], and ResNet-50 [3]
Resource availability:	https://github.com/Shahriar-Shakir-Sumit/ResTiNet.

## Method details

### ReSTiNet for low memory devices

**Fig. 1:**
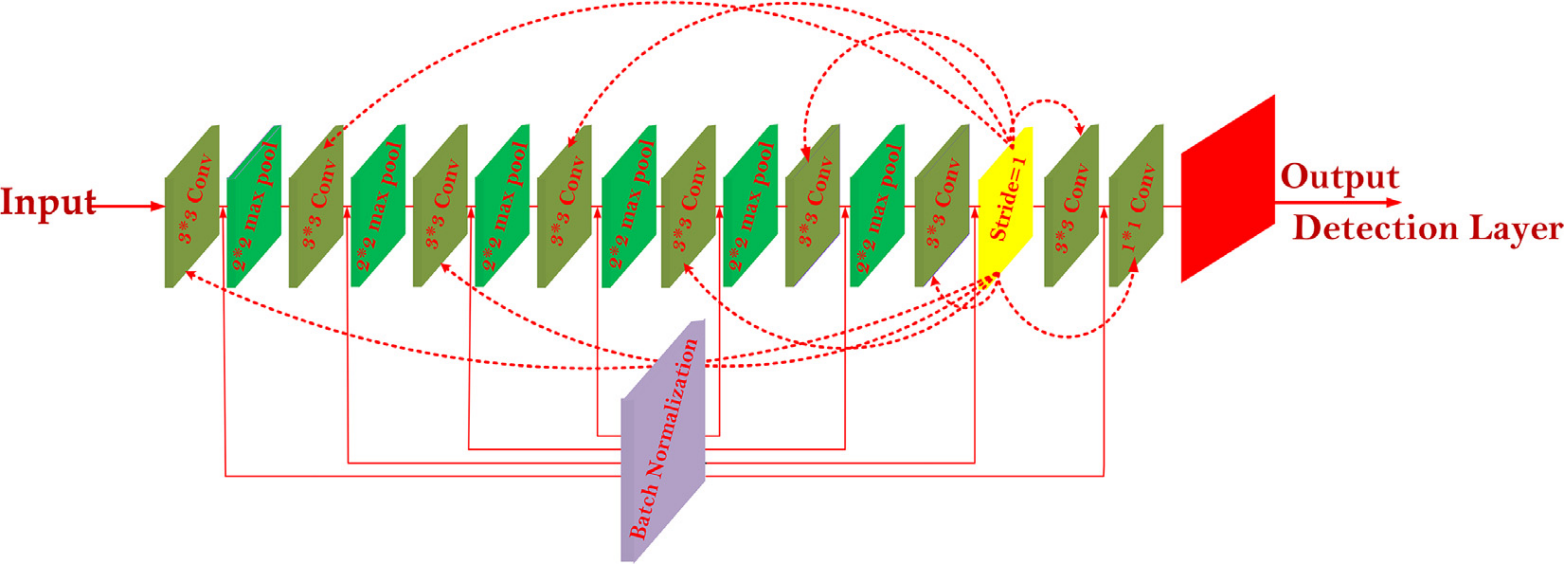
The architecture of Tiny-YOLO.

Fig. 1 illustrates the architecture of the Tiny-YOLO framework. This system consists of a detection layer, nine convolutional layers, and six max-pooling layers used to extract image features. This framework uses convolutional layers with 512 and 1024 filters, resulting in a large number of parameters, a large amount of memory, and slower recognition speed. The poor recognition accuracy of Tiny-YOLO is another problem. The arbitrary compression methods of the framework can further reduce detection accuracy.

**Fig. 2:**
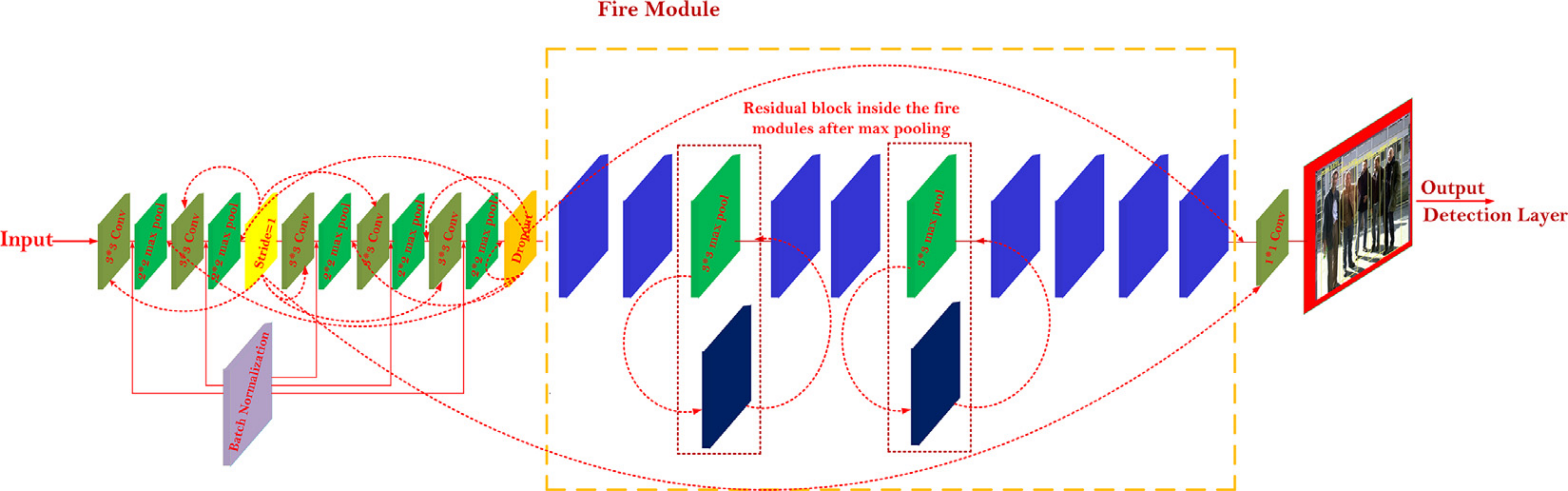
The architecture of ReSTiNet. Fire modules are adopted from SqueezeNet, which shrinks the model. Then, residual connections are integrated from the ResNet-50 network inside the fire modulesto enhance the proposed ReSTiNet's efficiency, reproduced from [11]. The experimental outcomes of ReSTiNet are available at [11].

To address these challenges, this study presents ReSTiNet, focusing on the performance of the framework's accuracy and the size of its weight. The proposed ReSTiNet framework is described in detail in Algorithm 1 and shown in Fig. 2.

**Algorithm 1:** ReSTiNet pseudocode.

1: ***Input:****Input(shape= (input_size, input_size,3))*
2: ***Input:****learning_ rate, epoch, batch_size* 3: ***Input:****iou_ threshold, score_ threshold* 4: ***Output:****output_shape, mAP*
5: ***def****fire_module(model, fire_id, squeeze, expand)*
6: ***def****maxpooling (pool_size, stride)*
7: ***def****resnet_block (model, filters, reps, stride)*
8: ***def****mAP (model):*
9: *map = model.evaluate (generator, iou_threshold,*
10: *score_threshold, average_precisions)*
11: ***return****map*
12: ***def****layer(conv, batchnorm, activation, maxpooling, dropout)*
13: ***def****main (){*
14: ***create layer1****: ([16,3,1], norm_1, leakyReLU[.1], 2, null)*
15: ***x→****layer1*
16: *for i in range(2,3,4,5):*
17: *create layer(i): ([32*^∗^*(2*^∗∗^*i), 3, 1], norm_ + str(i+2),*
18: *leakyReLU[.1], 2, [0.20])*
19: **x** (x) (layer(i))
20: //return **x**
21: *create fire_module1: (x, 2, 16, 64)*
22: *create fire_module2: (x, 3, 16, 64)*
23: *create maxpooling1: (3, 2)*
24: *create resnet_block1: (x, 64, 3, 1)*
25: *create fire_module3: (x, 4, 32, 128)*
26: *create fire_module4: (x, 5, 32, 128)*
27: *create maxpooling2: (3, 2)*
28: *create resnet_block1: (x, 128, 4, 2)*
29: *create fire_module5: (x, 6, 48, 192)*
30: *create fire_module6: (x, 7, 48, 192)*
31: *create fire_module7: (x, 8, 64, 256)*
32: *create fire_module8: (x, 9, 64, 256)*
33: dropout **→** 0.50
34: ***return****mAP(x), output_shape(x)}*

The objective of ReSTiNet is to design a framework that is faster, smaller and better capable of identifying people on low-memory devices. Instead of indiscriminately demolishing the convolution layers, network optimization is accomplished by reducing parameters to a reasonable level. The fire module in SqueezeNet compacts the network by employing a network bottleneck layer and expands the framework module without reducing the accuracy of detection remarkably. As a consequence, the fire module was implemented in order to accomplish the performance of a swifter as well as a tinier framework design. ReSTiNet thereafter searches for ways to maximize the detection accuracy while con- currently reducing the parameters. In [4], the authors obtained improved accuracy with fewer parameters by integrating residual blocks inside fire modules in the VGG-16 framework. As a result, the residual connection, which is employed by ReSTiNet to increase the accuracy of detection, is located inside the fire modules.

### Construction of ReSTiNet

Fig. 2 illustrates the ReSTiNet framework. ReSTiNet retains the first five frontal convolutional layers of Tiny-YOLO. The fire modules replace the layers along with 512 as well as 1024 filters in the Tiny-YOLO, which makes the model smaller. The suggested method is then assisted to obtain greater mAP by the integration of residual connections from the Resnet-50 framework within the fire modules. This study combines three popular frameworks: Tiny-YOLO [1,5], SqueezeNet [2], and the ResNet-50 [3,6] technique. The accomplishments are detailed as follows.

### Tiny-YOLO

A famous approach known as Tiny-YOLO, a diminutive of You Only Look Once (YOLO), was developed to produce a single-step system that combined the detection and classification strategies [1,7]. The bounding box and class predictions are generated based on a single evaluation of the input image.

This approach differs from traditional methods in that it predicts both the class and the bounding box simultaneously. The steps are as follows: The input image is divided over the *S* × *S* grid at the first step. Next, a confidence score including the relevant bounding box is given to each grid cell. The confidence score is the likelihood or possibility that the object is present in each bounding box and is mathematically determined by the Eq. (1).

(1) $$C=\text{Pr}\left(\text{Object}\right)*{\text{IOU}}_{pred}^{truth}\;$$

where, IOU (“intersection over union”) is detailed as a fraction that falls between 0 and 1 numerically. The intersection is the region where the bounding box predictor and the ground truth overlap. The union refers to the area that lies between the predictor and the ground truth. In ideal conditions, the IOU should be closer to 1, indicating that the ground truth is roughly equivalent to the bounding box prediction. In the same way, each grid cell can predict the conditional class probability *C* while the bounding boxes are being made. As a result, the class-specific probability function for each cell is described as,

$$\text{Pr}\left(\text{Clas}s_{i}|\text{Object}\right)*\text{Pr}\left(\text{Object}\right)*{\text{IOU}}_{pred}^{truth}=\text{Pr}\left(\text{Clas}s_{i}\right)*{\text{IOU}}_{pred}^{truth}$$

### Fire module of ReSTiNet

The purpose of introducing the fire module into ReSTiNet was to reduce the number of parameters while increasing the framework's depth and width. This action was taken to ensure the detection accuracy. This network has both the squeeze and expand components, which cause the framework to compress and expand. The squeeze or compress component employs NIN's convolutional layer with 1 × 1 size as a replacement for the standard layer with 3 × 3 sizes. The framework that uses the 1×1 strategy was determined to be more effective in terms of reducing the parameter numbers.

In addition, the detection accuracy does not change much because there is only one variable to learn about in the training parameter. Throughout the expansion, both 1×1 and 3×3 strategies generally are utilized. Finally, the concatenation layer concatenates the arriving outputs from the several convolutional layers.

**Fig. 3:**
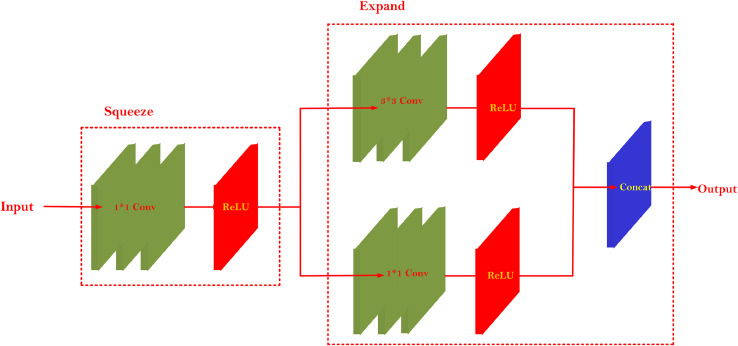
The structure of Fire Module. It is made up of two layers: Squeeze and Expand. Squeeze layer consists of a small number 1 × 1 filters and expand layer consists of a small number of 3 × 3 and 1 × 1 filters.

For a convolutional layer, the parameters are given as $c_{i}$ the number of channel input variables, *k* as the kernel size, and $c_{o}$ as the number of channel output variables. Using Eq. (2), the value of the number of parameters for the convolutional layer is then calculated. The number of channel inputs is *c_i_* for the fire module; $k_{s_{1}}$ is the kernel size of the squeeze component, and *s*_1_ is the number of channel output variables. If the value of $k_{s_{1}}$ is assigned to 1, a reduction of a large number of model parameters for the squeeze component is possible. The number of channel input variables is *s*_1_ followed by the kernel sizes $k_{e_{1}}$ and $k_{e_{3}}$ for the expand component. The total number of channel output variables is the sum of $e_{1}$ and $e_{3}$. Using Eq. (3), the number of model parameters is calculated. Fig. 3 illustrates the structure of the fire modules in ReSTiNet.

(2) $$P_{conv}=\;\left(c_{i}\;\times \;k^{2}+1\right)\;\times \;c_{o}$$

(3) $$\;P_{fire}=\left(c_{i}+\;k_{s_{1}}^{2}+1\right)\times \;S_{1\;}+\;\left(s_{1}+\;k_{e_{1}}^{2}+1\right)\;\times \;e_{1}+\;\left(s_{1}+\;k_{e_{3}}^{2}+1\right)\;\times \;e_{3}$$

The efficiency of the fire modules to be used more effectively is determined by their placement inside the framework. The ReSTiNet framework consists of eight fire modules in total. In the ReSTiNet framework, the 6th layer, which contains 512 filters, is substituted by the first four fire modules followed by a down-sampling strategy. The 7th and 8th layers, which have 1024 filters, are substituted by extra four fire modules prior to the 1 ×1 convolutional layer and detection layer. However, the selection of the input channel numbers $c_{i}$ is not constrained, although selecting a high input channel number would result in a parameter reduction.

### Residual block between the fire module

The optimization trajectory will have a negative slope because of the degradation when it is assumed that depth will improve the accuracy of detection. It has been noticed that the error rate in deep CNN designs is often greater than in other conventional neural ar- chitectures [4]. In [3], the authors developed a solution for degradation that allows certain stacked layers to accept the preexisting residual mapping. This is the region in which usual degradation prevents the layers from being consistent with the conventional subsidiary mapping. Eq. (5) indicates the subsidiary mapping as opposed to Eq. (4), where *H*(*x*) represents the desired mapping and *F* (*x*) describes the learned resid- ual mapping. The existent mapping has been changed to *F* (*x*) + *x*. In [3], the authors discovered that optimizing a residual-based mapping was easier than the main mapping.

(4) F(x)=H(x)

(5) F(x)≔H(x)−x

(6) H(x)=F(x)+x

However, one or more layers were not taken into account during the shortcut connections as stated in [3,4]. Eq. (6) defines the shortcut connections [3]. In [3], shortcut connections were used to perform identity mappings. The outputs of stacked layers are fused with the output values of shortcut connections. The latter has the benefit of permit- ting parameter-free calculation, utilizing minor values in the process. In [8], the authors designed a highway-based architecture by combining shortcut connections and gating functions with their parameters. The potential for optimization using stochastic gradient descent (SGD) is an additional advantage of shortcut connections [3]. Deep learning open-source libraries facilitate the integration of identity shortcut connections [3,4].

**Fig. 4:**
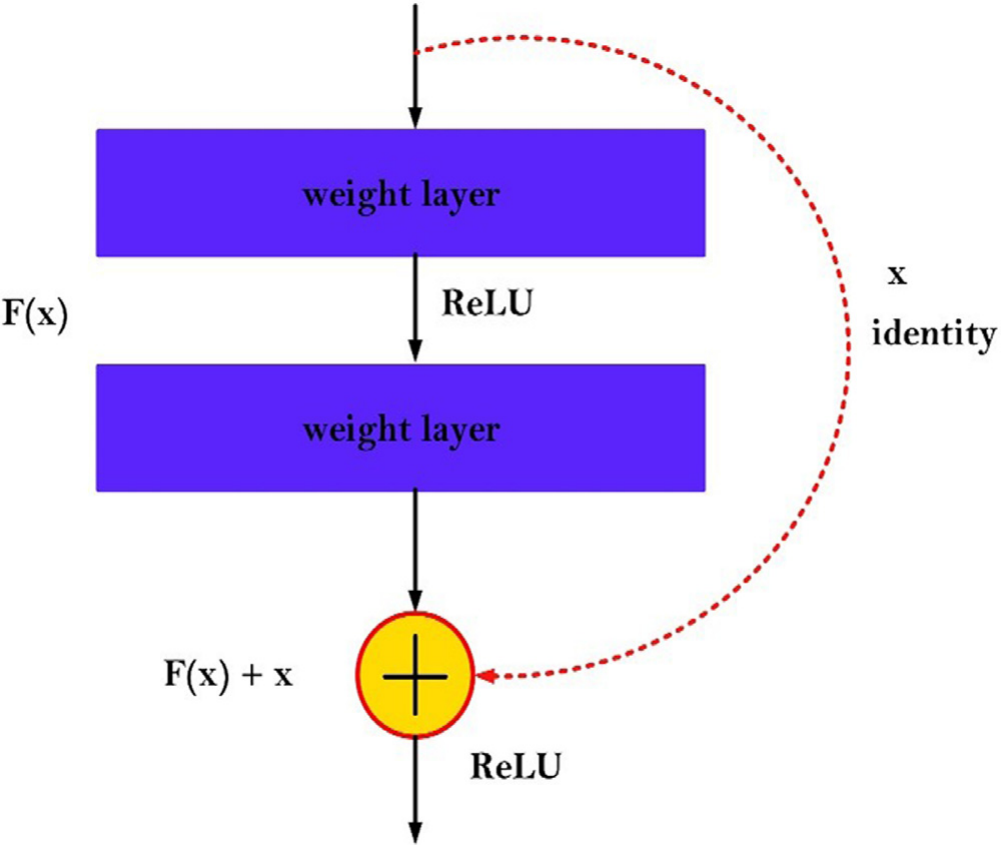
The structure of a residual block.

We have incorporated residual learning from ResNet-50 method into the ReSTiNet framework, followed by the down-sampling technique after the second and fourth fire modules. Building residual block is described in Eq. (7).

(7) $$y=F(x,W_{i})+x$$

where, *x* and *y* denote the input and output vectors of the layers, accordingly. The function *F* (*x, Wi*) represents the residual mapping that needs to be learned. As shown in Fig. 4, there are two layers, *F* = *W*_2_*_σ_*(*W*_1_*x*), in which *sigma* indicates the ReLU function and several biases were eliminated to reduce the notation complexity. The operation *F* + *x* was accomplished applying a shortcut connection and elemental-wise addition procedure in conjunction with the second ReLU (non-linearity) function. The shortcut connections in Eq. (7) do not increase the number of parameters or the computational complexity [3].

### Dropout in ReSTiNet

The mask that counteracts the actions of neurons in the subsequent layer is known as the dropout layer. This mask results in balancing neurons and maintains the integrity of the others. This layer is essential during CNN training because it mitigates the effects of over-fitting to the training data. Alternatively, there will be an impact from the initial data batches on the learning, resulting in disproportional performance outcomes. Therefore, the performance of learning the properties will be greatly decreased, and the delivery of such findings will be further delayed [9]. The standard approach for dropout is to employ a modest number between 20-50 percent of neurons, with 20 percent being a good starting point. A number that is too high prevents the framework from fully learning, while a value that is too low has no effect [10]. The recommended ReSTiNet framework uses 20% dropout in the convolutional layers (2nd–5th) and 50% dropout after the fire module to overcome the over-fitting issue.

### Loss function of ReSTiNet

In this research, we use a custom loss function like Tiny-YOLO, which has three parts: error in prediction coordinate, error in IOU, and classification error. The coordinate prediction error is defined as:

(8) $$\begin{array}{ccc} Error_{coord} & = & \lambda_{coord\;}\;\sum_{i=0}^{s^{2}}\;\sum_{j=0}^{B}L_{ij}^{obj}\left[{\left(x_{i\;}-x_{i}^{\wedge }\right)}^{2}+\;{\left(y_{i\;}-y_{i}^{\wedge }\right)}^{2}\right]\\ & & +\;\lambda_{coord\;}\;\sum_{i=0}^{s^{2}}\;\sum_{j=0}^{B}L_{ij}^{obj}\left[{\left(\sqrt{w_{i}}-\sqrt{w_{i}^{\wedge }}\right)}^{2}+{\left(\sqrt{h_{i}}-\sqrt{h_{i}^{\wedge }}\right)}^{2}\;\right] \end{array}$$

where $s^{2}$ represents the grid cell number of all scale. B denotes the bounding boxes number for every grid. $L_{ij}^{obj}$ represents target of the *i*-th grid cell which falls in the *j*-th bounding box. ($x_{i}^{\wedge }$*,* $x_{i}^{\wedge }$, $w_{i}^{\wedge }$, $\;h_{i}^{\wedge }$) and ($x_{i\;}$*,* $y_{i\;},$ $w_{i},\;h_{i}$) define the center coordinate, height and width of the predicted box and the ground truth, respectively.

The IOU error is defined with Eq. (9).

(9) $$\begin{array}{ccc} Error_{IOU} & = & \lambda_{coord\;}\;\sum_{i=0}^{s^{2}}\;\;\sum_{j=0}^{B}L_{ij}^{obj}{\left(C_{i\;}-C_{i}^{\wedge }\right)}^{2}\\ & & +\;\lambda_{noobj}\;\sum_{i=0}^{s^{2}}\sum_{j=0}^{B}L_{ij}^{noobj}\;{\left(C_{i\;}-C_{i}^{\wedge }\right)}^{2} \end{array}$$

where $C_{i}^{\wedge }$ and $C_{i\;}$ denote the predicted and true confidence, respectively.

The classification error is expressed as:

(10) $${Error}_{cls}=\sum_{i=0}^{s^{2}}L_{i}^{obj}\;\;\sum_{c\;classes}{\left(P_{i\;}\left(c\right)-P_{i}^{\wedge }\left(c\right)\right)}^{2}$$

where, $P_{i}^{\wedge }\left(c\right)$ defines the predicted value, while $P_{i\;}\left(c\right)$ defines the target true probability.

From the above equations, the resulting loss function is depicted in Eq. (11):

(11) $$\begin{array}{ccc} Loss & = & Error_{coord\;}+\;Error_{IOU\;}+\;Error_{cls}\\ & = & \lambda_{coord\;}\;\sum_{i=0}^{s^{2}}\sum_{j=0}^{B}L_{ij}^{obj}\;\left[{\left(x_{i\;}-x_{i\;}\right)}^{2}+\;{\left(y_{i\;}-y_{i\;}\right)}^{2}\right]\\ & & +\;\lambda_{coord\;}\;\sum_{i=0}^{s^{2}}\;\sum_{j=0}^{B}L_{ij}^{obj}\left[{\left(\sqrt{w_{i}}-\sqrt{w_{i}^{\wedge }}\right)}^{2}+{\left(\sqrt{h_{i}}-\sqrt{h_{i}^{\wedge }}\right)}^{2}\;\right]\\ & & +\;\lambda_{coord\;}\;\sum_{i=0}^{s^{2}}\sum_{j=0}^{B}L_{ij}^{obj}{\left(C_{i\;}-C_{i}^{\wedge }\right)}^{2}\;\\ & & +\;\lambda_{noobj}\;\sum_{i=0}^{s^{2}}\sum_{j=0}^{B}L_{ij}^{noobj}{\left(C_{i\;}-C_{i}^{\wedge }\right)}^{2}\;\\ & & +\;\sum_{i=0}^{s^{2}}L_{i}^{obj}\;\;\sum_{cclasses}{\left(P_{i\;}\left(c\right)-P_{i}^{\wedge }\left(c\right)\right)}^{2} \end{array}$$

### Time complexity, success and challenge of ReSTiNet

This section details the time complexity and success/challenge of the proposed ReSTiNet.

### Time complexity of the ReSTiNet

Some actions in the recommended method occur just once and have a time complexity of *O*(1). However, in the ReSTiNet several techniques have iteration and their time complexity is *O*(*n*^2^). Hence, the time complexity of our developed algorithm: *O*(1) + *O*(*n*^2^) = *O*(*n*^2^). Because of this, we describe this method as possessing a Quadratic Time Complexity, which means that as the quantity of the input rises, so does the amount of time required to execute it.

### Benefit of the ReSTiNet

This recommended approach is fairly flexible; therefore, it may be used to compress the many deep CNN models currently available. As human detection is the initial step in various applications, this proposed approach can be utilized for pedestrian detection and pose estimation on devices with limited memory.

### Limitation of the ReSTiNet

ReSTiNet uses the fire modules, which minimizes the model parameters and, therefore, the computing expenses. However, a substantial amount of computing still needs to be done to complete the task on a low-memory device. Therefore, the network is still trained on a machine (i.e., a remote server) that is capable of dealing with this computationally expensive procedure.

## Funding

YUTP-FRG (Cost Centre 015LCO-242), Universiti Teknologi PETRONAS (UTP) funded this work.

## CRediT authorship contribution statement

**Shahriar Shakir Sumit:** Conceptualization, Methodology, Software, Data curation, Investigation, Writing – original draft, Writing – review & editing. **Dayang Rohaya Awang Rambli:** Conceptualization, Methodology, Validation, Investigation, Writing – review & editing, Supervision, Funding acquisition. **Seyedali Mirjalili:** Conceptualization, Methodology, Validation, Investigation, Writing – review & editing, Supervision. **M. Saef Ullah Miah:** Software, Data curation, Validation, Investigation, Writing – review & editing. **Muhammad Mudassir Ejaz:** Software, Data curation, Validation, Investigation, Writing – review & editing.

## Declaration of Competing Interest

The authors declare that they have no known competing financial interests or personal relationships that could have appeared to influence the work reported in this paper.

## Data Availability

Data will be made available on request.

## References

[1] Redmon J., Divvala S., Girshick R., Farhadi A. (2016). Proceedings of the IEEE Conference on Computer Vision and Pattern Recognition.

[2] F. N. Iandola, S. Han, M. W. Moskewicz, K. Ashraf, W. J. Dally, and K. Keutzer, “Squeezenet: Alexnet-level accuracy with 50x fewer parameters and< 0.5 mb model size,” arXiv preprint arXiv:1602.07360, 2016.

[3] He K., Zhang X., Ren S., Sun J. (2016). Proceedings of the IEEE Conference on Computer Vision and Pattern Recognition.

[4] Qassim H., Verma A., Feinzimer D. (2018). Proceedings of the IEEE 8th Annual Computing and Communication Workshop and Conference (CCWC).

[5] Jiang P., Ergu D., Liu F., Cai Y., Ma B. (2022). A review of yolo algorithm developments. Procedia Comput, Sci,.

[6] Wen L., Li X., Gao L. (2020). A transfer convolutional neural network for fault diagnosis based on resnet-50. Neural Comput. Appl..

[7] Carion N., Massa F., Synnaeve G., Usunier N., Kirillov A., Zagoruyko S. (2020). Proceedings of the European Conference on Computer Vision.

[8] Hochreiter S., Schmidhuber J. (1997). Long short-term memory. Neural Comput..

[9] Srivastava N., Hinton G., Krizhevsky A., Sutskever I., Salakhutdinov R. (2014). Dropout: a simple way to prevent neural networks from overfitting. J. Mach. Learn. Res..

[10] Rennie S.J., Goel V., Thomas S. (2014). Proceedings of the IEEE spoken language technology workshop (SLT).

[11] Sumit S.S., Awang Rambli D.R., Mirjalili S., Ejaz M.M., Miah M.S.U. (2022). Restinet: On improving the performance of tiny-yolo-based cnn architecture for applications in human detection. Applied Sciences.

